# Microstructural Evolution and Mechanical Properties in Superlight Mg-Li Alloy Processed by High-Pressure Torsion

**DOI:** 10.3390/ma11040598

**Published:** 2018-04-13

**Authors:** Qian Su, Jie Xu, Yuqiao Li, Jae Ik Yoon, Debin Shan, Bin Guo, Hyoung Seop Kim

**Affiliations:** 1 Key Laboratory of Micro-Systems and Micro-Structures Manufacturing of Ministry of Education, Harbin Institute of Technology, Harbin 150080, China; 14B909052@hit.edu.cn (Q.S.); liyuqiaohit@126.com (Y.L.); shandebin@hit.edu.cn (D.S.); guobin@hit.edu.cn (B.G.); 2 School of Materials Science and Engineering, Harbin Institute of Technology, Harbin 150001, China; 3 Department of Materials Science and Engineering, Pohang University of Science and Technology, Pohang 37673, South Korea; suq33023@gmail.com

**Keywords:** microstructure, mechanical property, ultrafine-grains, high-pressure torsion, Mg-Li alloys

## Abstract

Microstructural evolution and mechanical properties of LZ91 Mg-Li alloy processed by high-pressure torsion (HPT) at an ambient temperature were researched in this paper. The microstructure analysis demonstrated that significant grain refinement was achieved after HPT processing with an average grain size reducing from 30 μm (the as-received condition) to approximately 230 nm through 10 turns. X-ray diffraction analysis revealed LZ91 alloy was consisted of α phase (hexagonal close-packed structure, hcp) and β phase (body-centered cubic structure, bcc) before and after HPT processing. The mean value of microhardness increased with the increasing number of HPT turns. This significantly increased hardness of specimens can be explained by Hall-Petch strengthening. Simultaneously, the distribution of microhardness along the specimens was different from other materials after HPT processing due to the different mechanical properties of two different phases. The mechanical properties of LZ91 alloy processed by HPT were assessed by the micro-tensile testing at 298, 373, 423, and 473 K. The results demonstrate that the ultra-fine grain LZ91 Mg-Li alloy exhibits excellent mechanical properties: tensile elongation is approximately 400% at 473 K with an initial strain rate of 1 × 10^−2^ s^−1^.

## 1. Introduction

Severe plastic deformation (SPD) is an effective method to produce ultra-fine grained (UFG) materials and it has received significant attention in the last two decades for the superior mechanical and physical properties produced in this way [1,2]. Typically, the grain size of materials processed by SPD is in the sub-micrometer or nanometer area. Some SPD processes are reported in the previous researches, such as the equal-channel angular pressing (ECAP) [3,4], high-pressure torsion (HPT) [5,6], and accumulative roll-bonding (ARB) [7,8,9]. Among them, HPT processing is particularly attractive due to the fact that processed grains are remarkably smaller than other techniques by experimental results [10,11,12]. The ultra-fine grain (UFG) materials processed by this method have excellent physical and mechanical properties, such as high intensity, good toughness, excellent ductility, and the potentiality of forming a superplastic at high strain rates [13].

Magnesium (Mg) alloys are widely used in the area of aerospace, railway, and automotive industries in view of their low density, high damping property, high cycle capacity, and lower energy demand [14,15]. However, the formability of magnesium alloys at ambient temperature is poor because of the limited slip systems of hcp structure [16]. Consequently, poor plasticity and cold formability at ambient temperature restrict their wide application [17,18]. Adding lithium (Li) into Mg alloys can reduce the axial ratio (c/a) of the hcp lattice and introduce phases with bcc structure which have more slip systems improving the deformation behavior effectively [19]. On the other hand, the strength of Mg alloys will be reduced due to the addition of Li element. In fact, Mg–Li alloy is the lightest metal structural material with high stiffness, excellent machinability, good magnetic-shielding, and resistance, whereas the strength of Mg–Li alloys is very low [20,21,22]. Usually, previous way to increase strength included addition of Zn, Al, or rare earth elements (RE); or addition of Sn with subsequent SPD and heat treatment. There is currently wide research activity on SPD processing of Mg alloys [23] including some research about grain refinement of HPT-processed Mg-Li alloys [24,25,26]. By describing the evolution of microstructure and micro-hardness after HPT processing, and the influence of HPT processing on the strength and ductility obtained from tensile testing at different temperatures, this paper provides detailed information about the HPT processing on LZ91 Mg-Li alloy and the subsequent mechanical performance when it was tensile tested at different temperatures.

## 2. Materials and Methods

**Figure 1:**
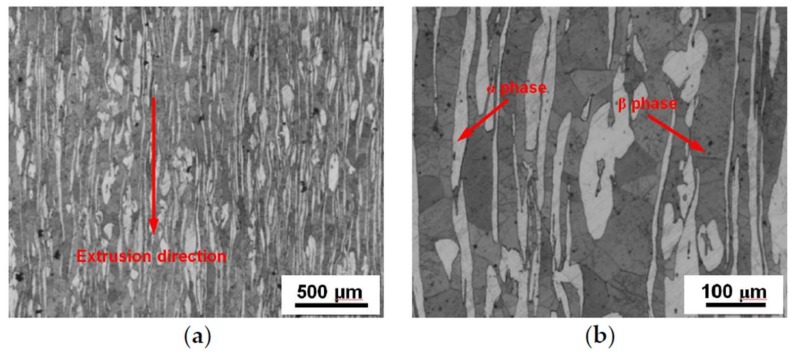
OM images of the as-received LZ91 alloy at (**a**) 50×, and (**b**) 200×.

Commercial LZ91 Mg-Li alloy rods with a diameter of 10.0 mm were used in this paper and the LZ91 alloy composition is shown in Table 1. Figure 1 shows the microstructure of the as-received LZ91 alloy observed by metallographic microscope (OLYMPUS, GX71, Olympus Company, Tokyo, Japan), where the white areas are α phase and the gray areas are β phase. The initial average grain size was approximately 30 μm from the result of Figure 1a. Meanwhile, both α phase and β phase were stretched along the extrusion direction and distributed homogeneously.

The LZ91 alloy rods were cut into disks with thicknesses of 1.5 mm first and then these disks were polished on two sides carefully before HPT processing. For processing of HPT, every disk was put in the depression of the lower anvil and then the two anvils of HPT processing machine were brought together with an imposed pressure. In this paper, all disks were processed with an imposed pressure (P) of 6.0 GPa at ambient temperature. Torsional straining was achieved by rotating the lower anvil with a rotation speed of 1 rpm. In addition, the rotation turns were r 1/4, 1, 5, and 10, respectively. Then these HPT processed disks were ground on SiC papers in series to 1200, and then the disks were mechanically polished to a mirror-like surface. 

X-ray diffraction (XRD) was carried out for the quantitative phase analysis on the LZ91 alloy before and after HPT processing with different numbers of turns. The radiation of XRD experiments was Cu Kα with a wave length λ = 0.15418 nm and the scanning range were 20° to 100°. To characterize the microstructure of LZ91 alloy processed by HPT with various numbers of turns, transmission electron microscopy (TEM, Tecnai G2 F30, FEI Company, Hillsboro, OR, USA) was carried out. The thin sections used in the experiment were obtained on a position of 2.5 mm from the center of the LZ91 alloy samples processed by HPT by ion thinning. 

Microhardness test was performed on the polished disks by a Shimadzu micro-hardness tester (Shimadzu, Kyoto, Japan) which was equipped with a Vickers indenter. The indentation load and dwell time of experiment are 100 gf and 10 s, respectively. The hardness of every disk was measured from the center along the radial direction with spacing of 0.5 mm between two indentations. 

**Figure 2:**
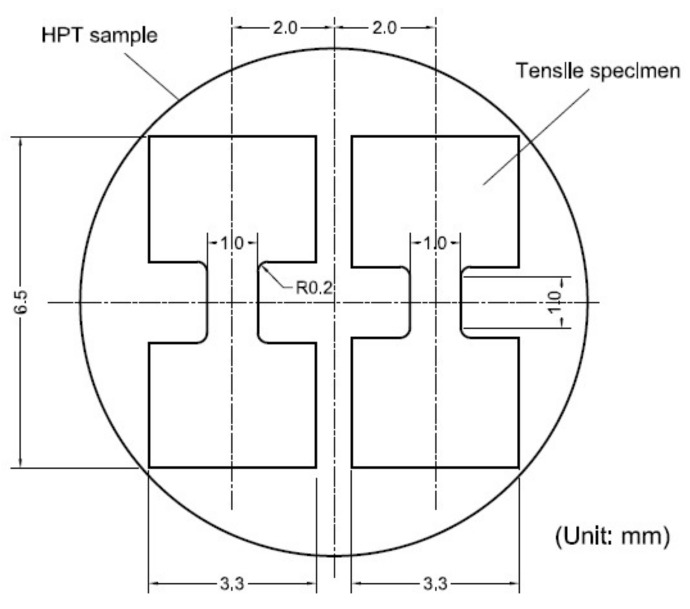
Sample size of micro-tensile testing.

The mechanical properties of the LZ91 alloy before and after HPT were determined using micro-tensile testing. The tensile samples were cut from disks using electro-discharge machining with symmetric off-center positions and the sample size was shown in Figure 2. Before the testing, tensile specimens were first ground on SiC papers in series to 1200# and then electropolished to remove swirl. The micro-tensile testing was performed at ambient temperature and elevated temperatures of 373, 423, and 473 K where the samples were heated by high temperature furnace with an initial stain rate of 1.0 × 10^−2^ s^−1^. 

## 3. Results

### 3.1. Microstructural Characteristics before and after HPT

**Figure 3:**
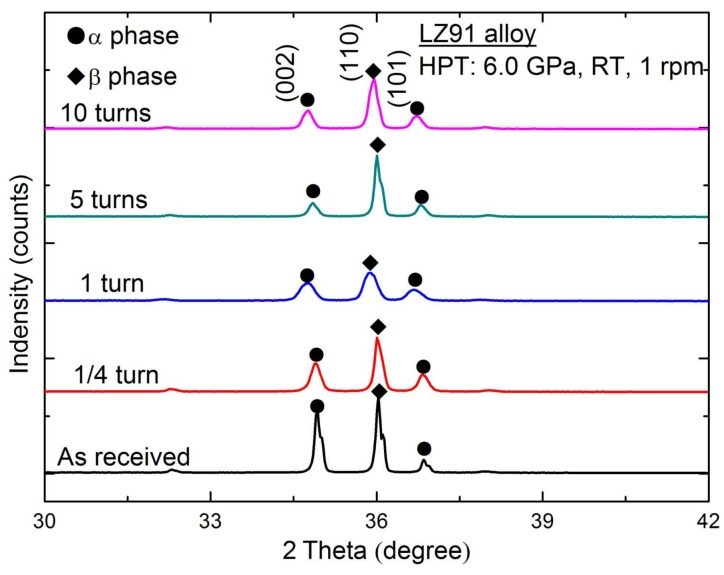
XRD patterns of LZ91 alloys before and after HPT processing.

The XRD spectrum presented in Figure 3 shows a quantitative phase analyses of the LZ91 alloy before and after HPT processing with different torsion turns. The results shown in Figure 1 reveal that the LZ91 alloy includes two different phases which are α phase (high content of Mg element) and β phase (high content of Li element). Meanwhile, there is no change in the phase composition of LZ91 alloy after HPT processing with different turns. 

**Figure 4:**
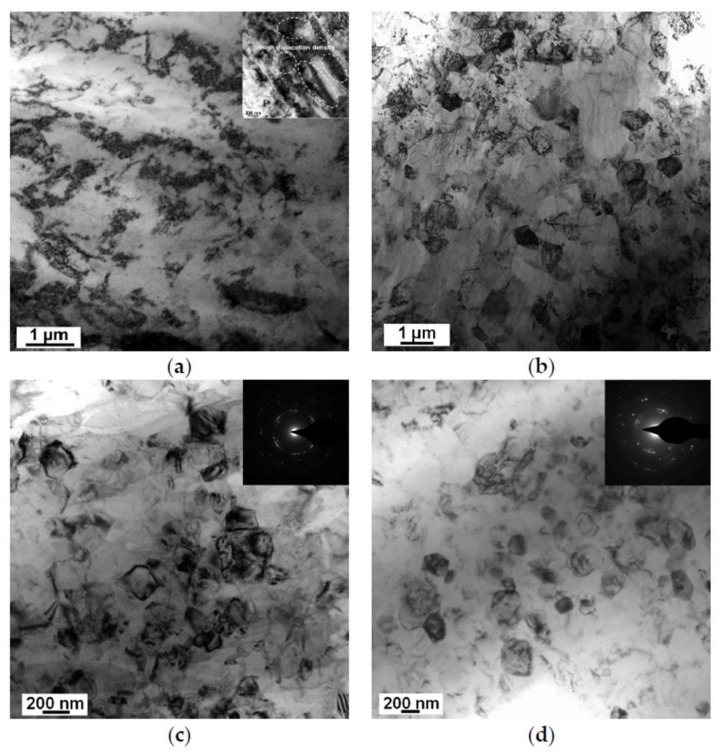
TEM images of LZ91 alloy after HPT processing with different turns of (**a**) 1/4; (**b**) 1; (**c**) 5; and (**d**) 10 at the one-half radius position.

**Figure 5:**
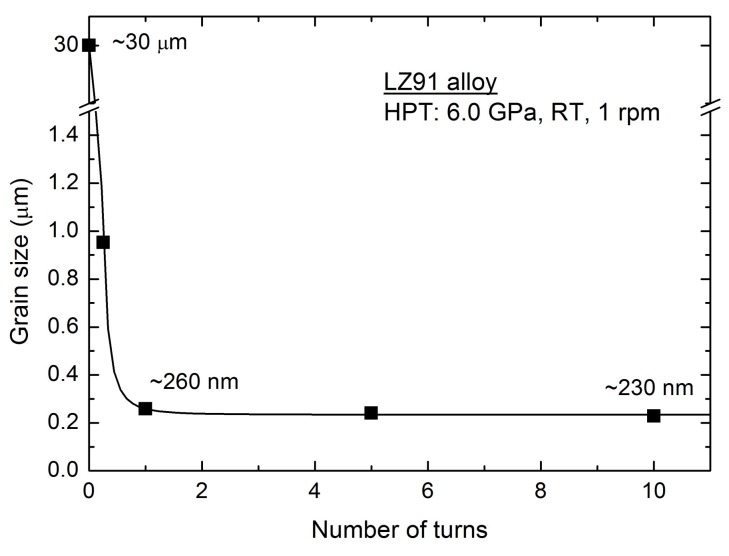
Average grain size of LZ91 alloy before and after HPT processing with increasing torsion turns.

Figure 4 shows the TEM micrographs of LZ91 alloy after HPT processing with different turns of a 1/4, b 1, c 5, and d 10 at the half-radius position. From the results, it can be seen that the grains of the LZ91 alloy were significantly refined (i.e., decreased from micron-scale to submicron-scale) after HPT processing. To express the evolution of microstructure, grain size evolution during HPT processing was shown in Figure 5.

In the early period of HPT processing, shear bands with a high dislocation density were formed because of severe plastic deformation. When the dislocation density reached a threshold value, the grain would disintegrate to subgrains, which were separated by small angle grain boundaries. With further processing of HPT, deformation occurred on shear bands which were located at previously unstrained parts of the material. The size of grain decreased steadily, and the shear bands coalesced. Thereafter, the small angle grain boundaries were displaced by large angle grain boundaries [27,28]. From the embedded TEM photograph in Figure 4a, a significant amount of dislocation pile-up, labeled by white elliptical regions, was formed. The average strain of atomic-level increased on account of the increasing of dislocation density. The selected area electron diffraction (SAED) patterns of Figure 4c,d show that the grain boundaries have high angles of misorientation [29,30]. Therefore, the grains of LZ91 alloy were refined significantly from ~30 μm to ~950 nm processed by HPT after 1/4 turn. During the period between 1/4 and 1 turn, the volume ratio of grain boundaries increased due to grain refinement, which resulted in the use of much more energy to continue the refining process. However, the input mechanical energy, part of which transforms into internal energy in the grains, is constant. Therefore, the refining rate declined. After 1 turn, the average grain size could reach about 260 nm. Meanwhile, dynamic balance was achieved between the grain refining caused by plastic deformation and grain growth induced by thermal effect [31]. Even so, the grains of the LZ91 alloy were refined slightly. After 10 turns, the grains were refined to 230 nm, although some nanocrystalline grains formed with size of ~90 nm, as shown in Figure 4d. This grain refinement is in line with the earlier researches of various magnesium alloys processed by HPT at room temperature [32,33,34,35,36,37,38,39,40], as documented in Table 2.

### 3.2. Microhardness Evaluation during HPT Processing

**Figure 6:**
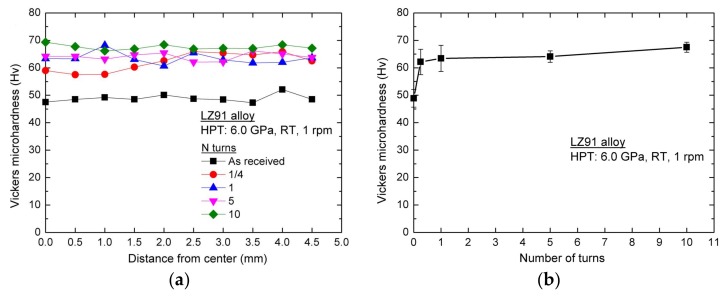
Vickers microhardness of LZ91 alloy (**a**) Along the radius of disk and (**b**) Average values before and after HPT processing with various number of turns.

Figure 6a shows the variation of Vickers microhardness along the radial direction of LZ91 alloy before and after HPT processing with 1/4, 1, 5, and 10 turns. Figure 6b shows the average values of Vickers microhardness shown in Figure 6a. The lower black line shows the initial microhardness for the original LZ91 alloy. These results reveal that the microhardness increased for the LZ91 alloy processed by HPT after 1/4 turn where the hardness increased from ~49 Hv to ~62 Hv, and the microhardness of LZ91 alloy processed by HPT became homogeneous after five turns. However, the variation of Vickers hardness along the radius of disk after HPT processing for LZ91 alloy is different from the previous reports. This difference may be caused by the existing of different two phases. 

As shown in Figure 4a,b, the microstructure of LZ91 alloy is inhomogeneous after 1/4 and 1 turn, and the two phases have different mechanical properties, so the distribution of microhardness for LZ91 alloy processed by HPT after 1/4 and1 turn is different from earlier reports. After 5 and 10 turns, the microstructure becomes homogeneous as shown in Figure 4c,d, and the influence of two different phases decreases, so the distribution of microhardness becomes homogeneous.

### 3.3. Tensile Behavior before and after HPT Processing

**Figure 7:**
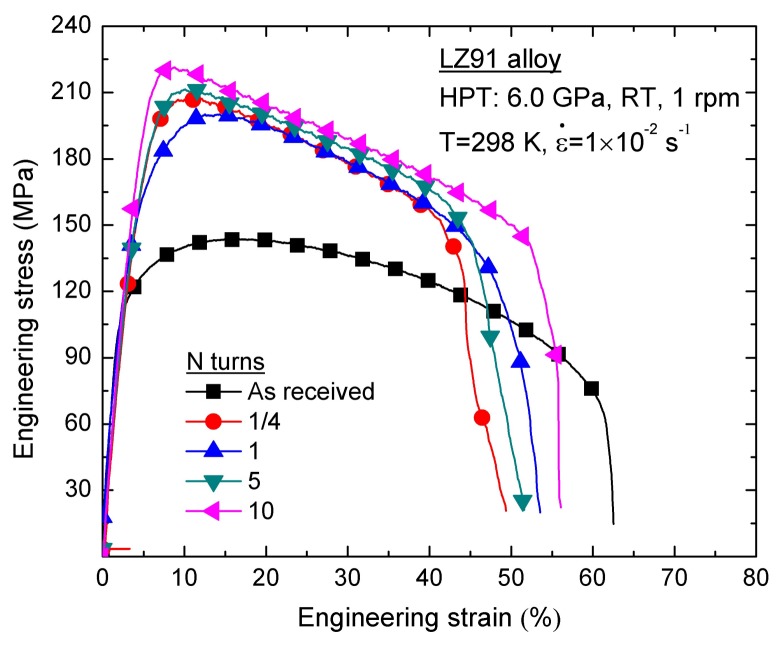
Engineering stress-engineering strain curves of LZ91 alloy before and after HPT processing through 1/4, 1, 5, and 10 turns at room temperature with an initial strain rate of 1 × 10^−2^ s^−1^.

Mechanical properties of the LZ91 alloy before and after HPT processing were evaluated using micro-tensile tests. Figure 7 shows the engineering stress-engineering strain curves of LZ91 alloy before and after HPT processing through 1/4, 1, 5, and 10 turns at room temperature with an initial strain rate of 1 × 10^−2^ s^−1^. The results showed that the yield stress obviously increased with increasing torsion turns of HPT at 298 K.

The yield stress of the LZ91 alloy processed by HPT could reach ~152 MPa after 10 turns, (1.7 times that of the as-received alloy). This result is mainly attributed to the Hall-Petch effect (i.e., grain size strengthening [41,42]), by which the strength increases with decreasing grain size. As analyzed in Section 3.1, the grains of LZ91 alloy were refined significantly from 30 μm to about 230 nm. Hence, the volume ratio of the grain boundary increased significantly, which would restrict the movement of dislocations across grain boundaries during plastic deformation at low temperature. Therefore, the yield stress of the specimens improved after HPT processing at low temperature, and this result is in line with the analysis in Section 3.2.

**Figure 8:**
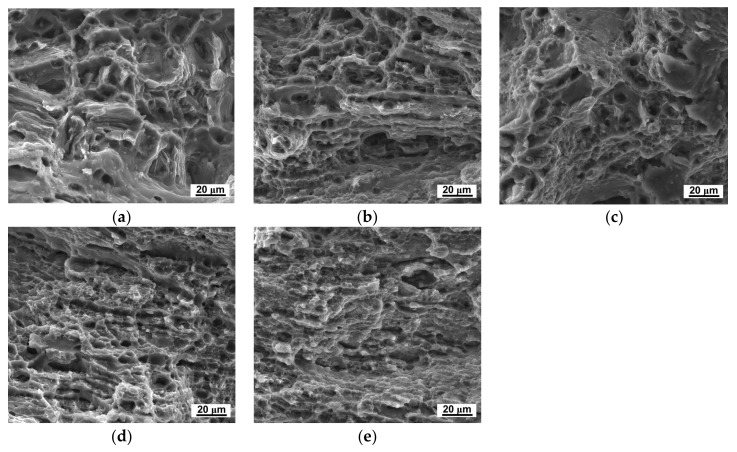
Micro-tensile fracture morphology of the LZ91 alloy (**a**) Before and after HPT processing through (**b**) 1/4; (**c**) 1; (**d**) 5, and (**e**) 10 turns.

In the plastic deformation processing, dislocation pile-up on grain boundaries is achieved. Therefore, the refined grains would also induce decrease of the ductility. As shown in Figure 7, the elongation to failure decreased after HPT processing. Figure 8 shows the micro-tensile fracture appearance fracture morphology of the LZ91 alloy before and after HPT processing, with different turns at 298 K. Before HPT processing, there were plenty of large, deep dimples, which accounted for the superior ductility of the extruded alloy as shown in Figure 8a. As shown in Figure 8b–e, the number of dimples decreased after HPT, and the dimples become small and shallow. Meanwhile, tearing ridges were observed in the fracture morphology. The evolution of the micro-tensile fracture morphology corresponds with the decrease of ductility.

**Figure 9:**
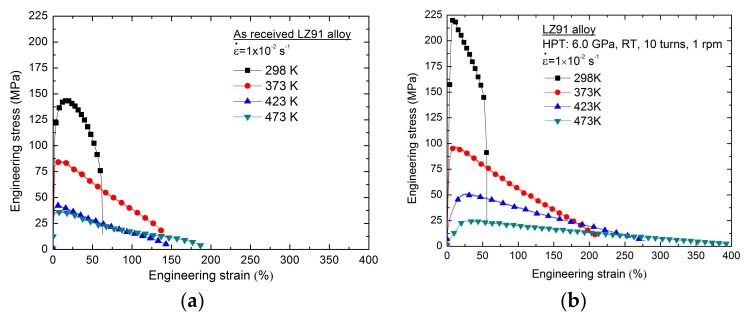
Engineering stress-engineering strain curves of LZ91 alloy (**a**) Before and (**b**) After HPT processing through 10 turns at different temperatures with a strain rate of 1 × 10^−2^ s^−1^.

Figure 9 shows the engineering stress-engineering strain curves of LZ91 alloy before and after HPT processing through 10 turns at different temperatures with an initial strain rate of 1 × 10^−2^ s^−1^. The results indicate that the yield strength decreases and elongation increases with increasing temperature of deformation. Meanwhile, the samples before and after HPT all exhibited high ductility at high temperature, and the plasticity after HPT processing was better than the original material. The measured elongation to failure of the LZ91 alloys before and after HPT processing with 10 turns from 298 K to 473 K using a strain rate of 1 × 10^−2^ s^−1^ is presented in Figure 9. As analyzed by the previous micro-tensile results at room temperature, there was a slight decrease of ductility after HPT processing. However, at elevated temperature the ductility improved, and the increase of ductility after HPT processing was more significant with increasing temperature. At a temperature of 473 K, the measured elongations to failure of LZ91 alloy after HPT processing with 10 turns could reach approximately 400% at an initial strain rate of 1 × 10^−2^ s^−1^. 

**Figure 10:**
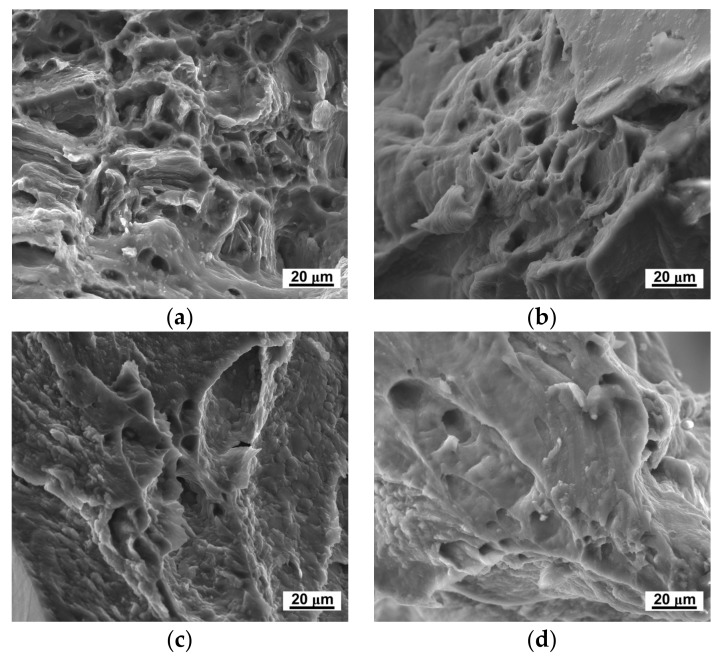
Micro-tensile fracture morphology of the original LZ91 alloy at temperature of (**a**) 298 K; (**b**) 373 K; (**c**) 423 K; and (**d**) 473 K.

**Figure 11:**
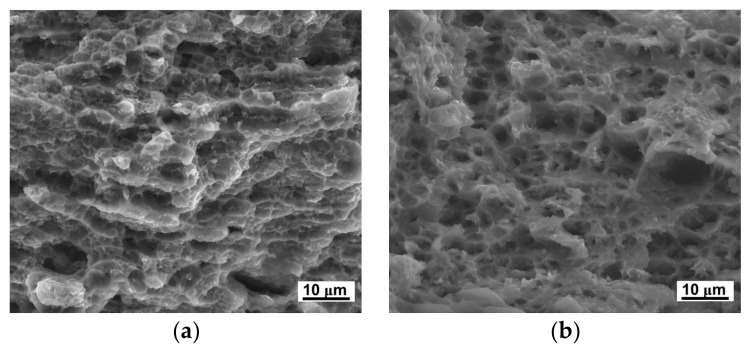

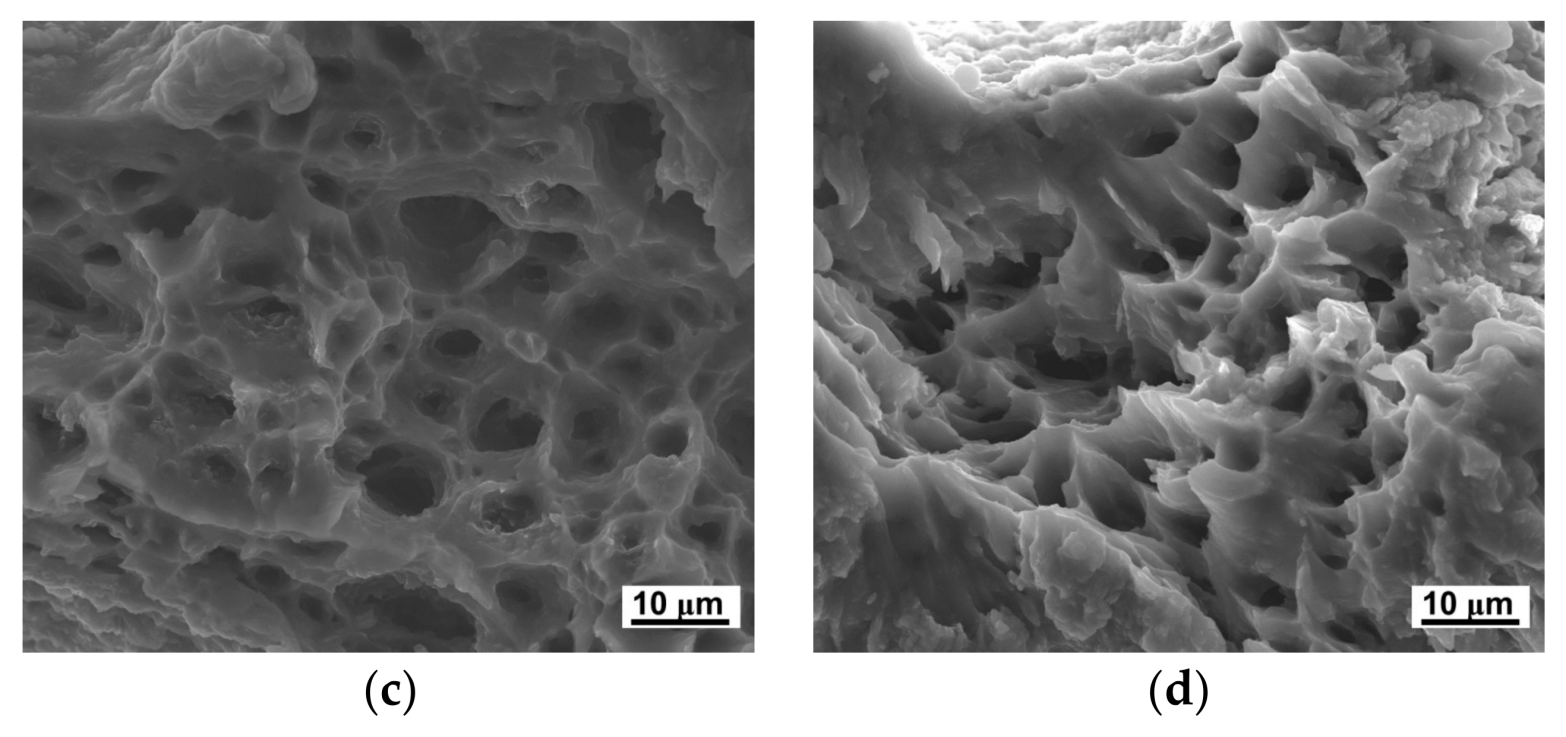
Micro-tensile fracture morphology of the LZ91 alloy processed by HPT through 10 turns at temperatures of (**a**) 298 K; (**b**) 373 K; (**c**) 423 K; and (**d**) 473 K.

The improvement of ductility at elevated temperature could mainly be attributed to the grain refinement. At elevated temperature, both the grain interior strength and grain boundary strength decreased; however, the grain boundary strength was more sensitive to temperature. At elevated temperature, the rotation and sliding of grains were easier in fine-grained materials. Therefore, the LZ91 alloy processing by HPT after 10 turns with an average grain size of ~230 nm presents excellent high-temperature ductility. Figure 10 and Figure 11 show the micro-tensile fracture morphology of LZ91 alloy before and after HPT processing through 10 turns at different temperatures. From these results, we can see that the number of dimples increased with increasing temperature, and the dimples became deeper and bigger. The evolution of the micro-tensile fracture morphology corresponds to the increase of ductility.

## 4. Discussion

### 4.1. Microstructural Evolution in the Mg-Li Alloy after Processing by HPT

The microstructural evolution of Mg-Li alloy during HPT processing indicates the possibility of preparing UFG LZ91 alloy with homogenous microstructure through HPT processing. From previous results, it can be seen that the average grain size of the LZ91 alloy was reduced significantly from ~30 μm to ~260 nm after 1 turn. However, after 1 turn, the refining rate declined due to part of input mechanical energy transforms into internal energy in the grains. After HPT processing through 5 turns, the microstructure of LZ91 alloy, with an average size of 240 nm, becomes homogeneity. In addition, the average grain size was reduced to 230 nm after 10 turns finally. In general, with the increasing torsion turns of HPT, the grain size decreases where the grain size changes from ~30 μm to ~230 nm after HPT processing through 10 turns at room temperature Therefore, it is confirmed that HPT processing is an effective and superior method to obtain an UFG Mg-Li alloy with singularly fine grains and homogeneous microstructure.

### 4.2. Microhardness Evolution in the Mg-Li Alloy after Processing by HPT

During HPT processing, the grain refining was achieved due to severe plastic deformation. The average grain size of LZ91 alloy decreased from ~30 μm (as received) to ~230 nm (after HPT processing through 10 turns). The improved microstructure with fine grain can contribute the increasing and homogeneity of hardness. After HPT processing through 10 turns, the average hardness across the disk is approximately 68 Hv as presented by Figure 6.

**Figure 12:**
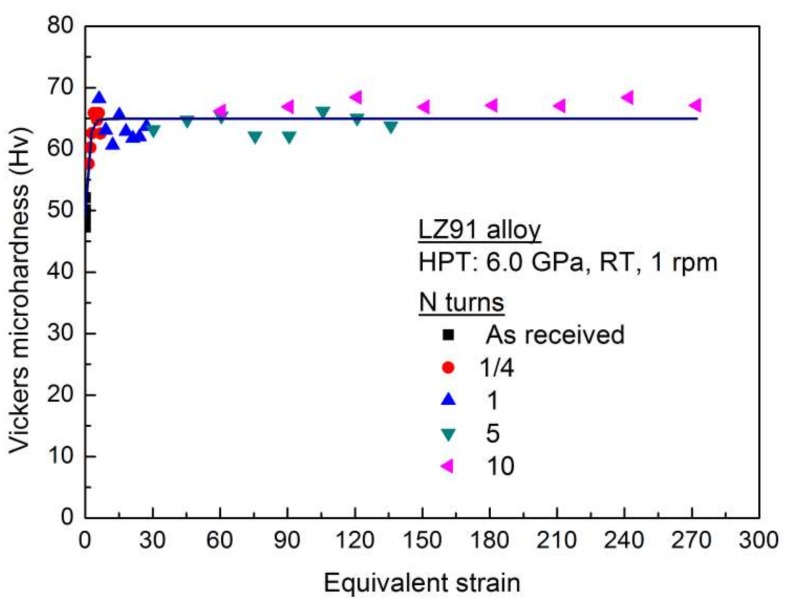
Relationship between Vickers microhardness and equivalent strain for LZ91 alloy before and after HPT processing with various numbers of torsion turns.

In practice, the relationship between the microhardness and the equivalent von Mises strain, *ε_eq_*, is given from the following expression [43,44,45]:

(1) $$\varepsilon_{eq}=\frac{2\pi Nr}{h\sqrt{3}},$$

where *r* is the radial range from the center of the specimen and *h* is the thickness of the specimen. Early experiments show that the microhardness value may be directly related to equivalent strain on HPT processing [46]. This correlation of this paper is shown in Figure 12 where all of the datum points are from Figure 7.

The severe grain refinement during HPT processing induces clear improvement in the hardness due to grain boundary strengthening. Additionally, with the increasing number of HPT turns, the hardness of HPT processed specimens improved. In addition, the regular increasing of hardness may be attribute to Hall-Petch strengthening when the relationship between hardness and grain size is reformulated as follows [47],

(2) $$H=H_{0}+k_{H}d^{-1/2}$$

where *H* is the hardness of specimen, *H*_0_ and *k_H_* are the appropriate material constants associated with the hardness measurements.

**Figure 13:**
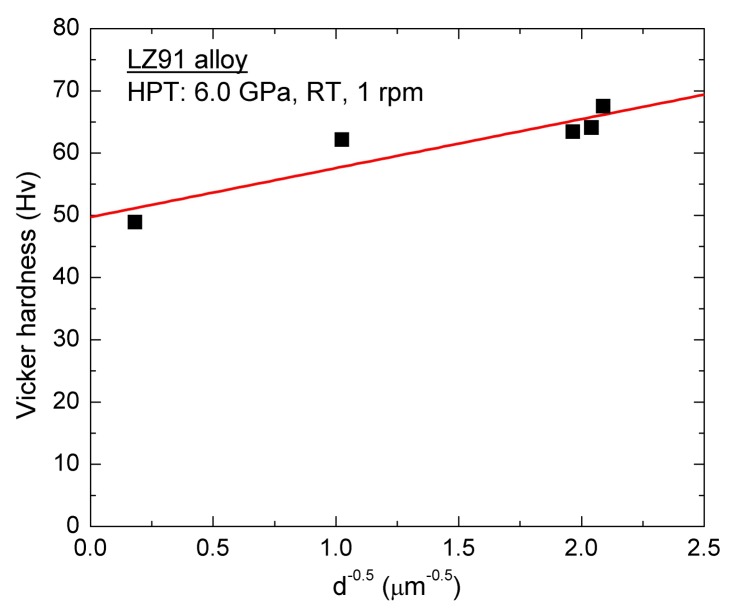
Relationship between Vickers microhardness and *d*^−1/2^ for the LZ91 alloy before and after HPT through 1/4, 1 5 and 10 turns at the one-half radius position.

The availability of using the Hall-Petch relationship to explain experimental results was demonstrated in earlier researches for other alloys after ECAP or HPT processing [47,48,49,50]. For the LZ91 alloy used in this paper, there is no precipitate-hardening and previous researches [26,47] indicate that the solution-hardening has little influence on the strengthening, so Hall-Petch relationship can be used to explain the experimental results in this paper. The Vickers microhardness results shown in Figure 6 were re-plotted as a function of *d*^−1/2^ for LZ91 alloy before and after HPT of 1/4, 1, 5, and 10 turns at the one-half radius position. The result is shown in Figure 13 and the result reveals that there has a conventional linearity relationship between hardness and *d*^−1/2^ for the present LZ91 alloy before and after HPT processing. As shown in the above investigations, the LZ91 alloy exhibited good Hall-Petch strengthening for grain refinement. Simultaneously, the microhardness of the HPT-processed specimens was homogeneous after five or more turns, which is in line with some earlier researches about the gradual improvement of hardness homogeneity for different materials processed by HPT [51,52,53]. Cumulative deformation of the materials processed by increased with the number of turns, and the material microstructure became uniform when the cumulative deformation increased to saturation values. For these reasons, the microhardness of LZ91 alloy processed by HPT becomes homogeneous after five or more turns.

### 4.3. The Potential Application of the UFG Mg-Li Alloy on Micro-Forming Technology

In related to micro-forming, the processing is converted from traditionally macroscopic level to micro level. Thus, the effect of grain size on the flow behavior and the overall formability becomes much more significant due to the inherent size effects [54,55]. Particularly, the uneven material performance would be brought about due to grain size effect, which can produce the deviation of the forming shape and lead to inhomogeneous properties of the products. A primary purpose of this study was to evaluate the potential applications of using the alloy in micro-forming for the UFG Mg-Li alloy using micro-tensile testing. As shown by Figure 7 and Figure 9, it was confirmed that during high temperature micro-tensile testing the flow stress decreased and the ductility was improved with increasing numbers of HPT turns. After 10 turns HPT processing, the ductility is improved significantly. The elongation to failure can reach about 400% at 473 K with an initial strain rate of 1.0 × 10^−2^ s^−1^ which is much higher than the value of as received LZ91 alloy.

These above results reveal that the UFG LZ91 alloy processing by HPT exhibits excellent formability at a relatively low temperature. Therefore, the UFG LZ91 alloy can be used in micro-forming processing with better formality compared with CG materials. Hence, there is potential application of the UFG LZ91 alloy on micro-forming based on present results. 

## 5. Conclusions

(1) The Mg-Li alloys were prepared via HPT processing with a pressure of 6.0 GPa up to 10 turns at ambient temperature. The average grain size diminished from ~30 μm (the original specimen) to ~230 nm (the HPT-processed specimen after 10 turns). The XRD results reveal the alloy was consist of hcp α-phase and bcc β-phase before and after HPT processing.
(2) Vickers microhardness measurements indicate the average microhardness increases significantly with increasing number of HPT turns. Meanwhile, after five or more turns, the microhardness of HPT-processed LZ91 alloy is homogeneous. This significantly increased hardness can be explained by Hall-Petch strengthening. The variation of Vickers hardness along the radius of disk after HPT processing for LZ91 alloy is different from the previous reports which can be explained by the existing of two different phases.
(3) The results from micro-tensile testing of the LZ91 alloy before and after HPT indicate that both the strength and ductility of LZ91 are improved with increasing number of HPT turns at both ambient and elevated temperatures. The maximum recorded tensile elongation is approximately 400% at 473 K with the initial strain rate of 1 × 10^−2^ s^−1^, indicating that after 10 turns HPT processing the ductility is improved significantly.
(4) Based on the experimental results, it is confirmed that the UFG LZ91 Mg-Li alloy processed by HPT processing after 10 turns presents enormous potential application to micro-forming.

## Figures and Tables

**Table 1:** The LZ91 alloy composition.

Element	Li	Zn	Mn	Else
Content (wt %)	8.92	0.97	0.1	/

**Table 2:** Grain sizes of magnesium and its alloys processed by HPT.

Material	HPT			Grain Size (nm)	Reference
	Turns	Pressure(GPa)	Temperature		
Mg-8% Li	5	3.0	RT	~500	Matsunoshita et al. [32]
Mg-8% Li	20	6.0	RT	~240	Edalati et al. [33]
Pure Mg	10	6.0	RT	~1000	Figueiredo et al. [34]
AZ31	5	6.0	RT	~900–1200	Huang et al. [35]
AZ31	10	6.0	RT	~110	Xu et al. [30]
AZ31	15	2.5	RT	~150–200	Stráská et al. [36]
ZK60	5	2.0	RT	~1000	Torbati-Sarraf et al. [37]
ZK60A	5	6.0	RT	~2000–5000	Lee et al. [38]
AZ80	10	6.0	RT	~200	Alsubaie et al. [39]
Mg-3.4 Zn	20	5.0	RT	~140	Meng et al. [40]
Mg-8.92% Li	10	6.0	RT	~230	Present paper
